# Mutual information minimization enhances noise robustness and induces fault containment in functionally differentiated recurrent neural networks

**DOI:** 10.3389/fnins.2026.1892212

**Published:** 2026-08-13

**Authors:** Yuki Tomoda, Yutaka Yamaguti

**Affiliations:** 1 Graduate School of Engineering, Fukuoka Institute of Technology, Fukuoka, Japan; 2 Faculty of Information Engineering, Fukuoka Institute of Technology, Fukuoka, Japan

**Keywords:** chaotic dynamics, functional differentiation, modularity, mutual information, recurrent neural network, robustness

## Abstract

Functional differentiation, the emergence of specialized neural populations, is a hallmark of biological brains and has been proposed to provide robustness, metabolic efficiency, and evolvability. In our previous work, we showed that minimizing mutual information (MI) between predefined subgroups of a recurrent neural network (RNN), using mutual information neural estimation (MINE), promotes the emergence of functionally specialized modules. However, whether such information-theoretically induced differentiation translates into functional benefits has remained unclear. Here we examine the robustness of MI-minimized RNNs trained on a chaotic signal separation task in which a superimposed input from the Lorenz and Rössler systems must be demixed into separate output channels. We find that MI minimization, while preserving task accuracy, consistently, and significantly enhances tolerance to input noise and produces qualitatively distinct responses to neuronal ablation: damage to the subgroup an output relies on impairs that output sharply, whereas damage to the other subgroup leaves it largely intact. This selectivity is essentially absent in control networks trained without the MI constraint. These results indicate that minimizing statistical dependence between neural populations not only induces functional specialization but also confers a form of fault containment, isolating damage to the affected output while sparing the others, supporting the view that modular organization is an adaptive consequence of information-theoretic constraints on neural representation.

## 1 Introduction

The mammalian brain exhibits a striking degree of functional differentiation, with distinct neural populations becoming specialized for specific computations (Sporns and Betzel, 2016; Meunier et al., 2010). A central question is how such modular organization arises. Self-organization has long been proposed as a guiding principle for this process; in particular, the self-organization-with-constraints framework of Tsuda et al. (2016) holds that functional modules need not be externally prescribed; instead, they can emerge through the interplay of intrinsic neural dynamics and internal constraints. This self-organized modularity has been argued to confer several adaptive advantages, including efficient information processing, evolvability, and graceful behavior under noise and partial damage (Kashtan and Alon, 2005; Clune et al., 2013). Yet the relationship between functional specialization and these robustness benefits has remained insufficiently characterized, particularly in computational models that produce specialization without hand-designed architectural priors.

Information-theoretic principles have long been invoked to explain self-organization in neural systems. Linsker (1988) introduced the InfoMax principle, in which network structure emerges from maximizing the mutual information between inputs and outputs; the same idea underlies information-maximization approaches to blind source separation (Bell and Sejnowski, 1995) and was later extended to recurrent networks as Recurrent InfoMax (Tanaka et al., 2009) and to evolutionary models of functional differentiation in coupled-map networks (Yamaguti and Tsuda, 2015; Watanabe et al., 2020). More recently, the information bottleneck framework recast deep representation learning as a trade-off between input compression and the retention of task-relevant information (Tishby and Zaslavsky, 2015), a proposal whose applicability to deep networks has since been actively debated (Saxe et al., 2019); in parallel, neural estimators of mutual information (Belghazi et al., 2018) have made such information-theoretic objectives directly optimizable in high-dimensional networks. A common thread across this body of work is the *maximization* of an information measure. A complementary possibility, central to the present study, is that *minimizing* mutual information between predefined sub-populations can itself act as an organizing principle, driving them to specialize and thereby giving rise to functional modularity.

Building on this perspective, our previous work (Tomoda et al., 2026) introduced a method to induce functional differentiation in recurrent neural networks (RNNs) by minimizing mutual information (MI) between predefined neural subgroups, using mutual information neural estimation (MINE) (Belghazi et al., 2018). We showed that this information-theoretic constraint promotes the emergence of functionally specialized modules, with functional modularity developing earlier and more prominently than structural modularity. These results identified MI minimization between sub-populations as an effective driver of self-organized functional differentiation in artificial networks. The *functional significance* of the resulting differentiation remained open, however; in particular, it was unclear whether it confers any of the robustness benefits long attributed to modular organization in biology.

In the present study, we characterize, for the first time, the robustness properties of RNNs trained with MI minimization on a chaotic signal separation task. All networks analyzed here were trained anew for this work following the identical protocol of our previous study, which did not examine robustness; the emergence of functional differentiation is reproduced on these networks as a control, and the noise-robustness and ablation analyses that follow are new. We evaluate both resistance to input noise and responses to neuronal ablation, the latter by comparing the removal of neurons from the subgroup most vs. least coupled to each output, in networks trained with and without the MI constraint. We find that MI minimization not only preserves task accuracy but also consistently enhances noise robustness and produces a qualitatively distinct ablation profile: damage to the subgroup an output relies on impairs that output sharply, whereas damage to the other subgroup leaves it largely intact, a selectivity that is essentially absent in networks trained without the MI constraint. These findings link information-theoretic constraints to a concrete adaptive function, namely fault containment, whereby damage is isolated to the affected output rather than spreading across the network. They support the view that modular organization in neural systems can be shaped by information-theoretic principles.

## 2 Materials and methods

The chaotic signal separation task and the network model follow our previous study (Tomoda et al., 2026), in which the same paradigm was used for the GRU-based experiments. Here we describe only the components essential for the present robustness analysis and refer the reader to Tomoda et al. (2026) for full implementation details. All networks analyzed in the present study were trained anew for this work, following the identical protocol of Tomoda et al. (2026); none of the analyses below reuse networks from that study. The functional-differentiation results are reproduced on these newly trained networks both for completeness and as a control for the robustness analyses, whereas the noise-robustness and fault-containment analyses that follow are new to this study.

### 2.1 Task: chaotic signal separation

**Figure 1:**
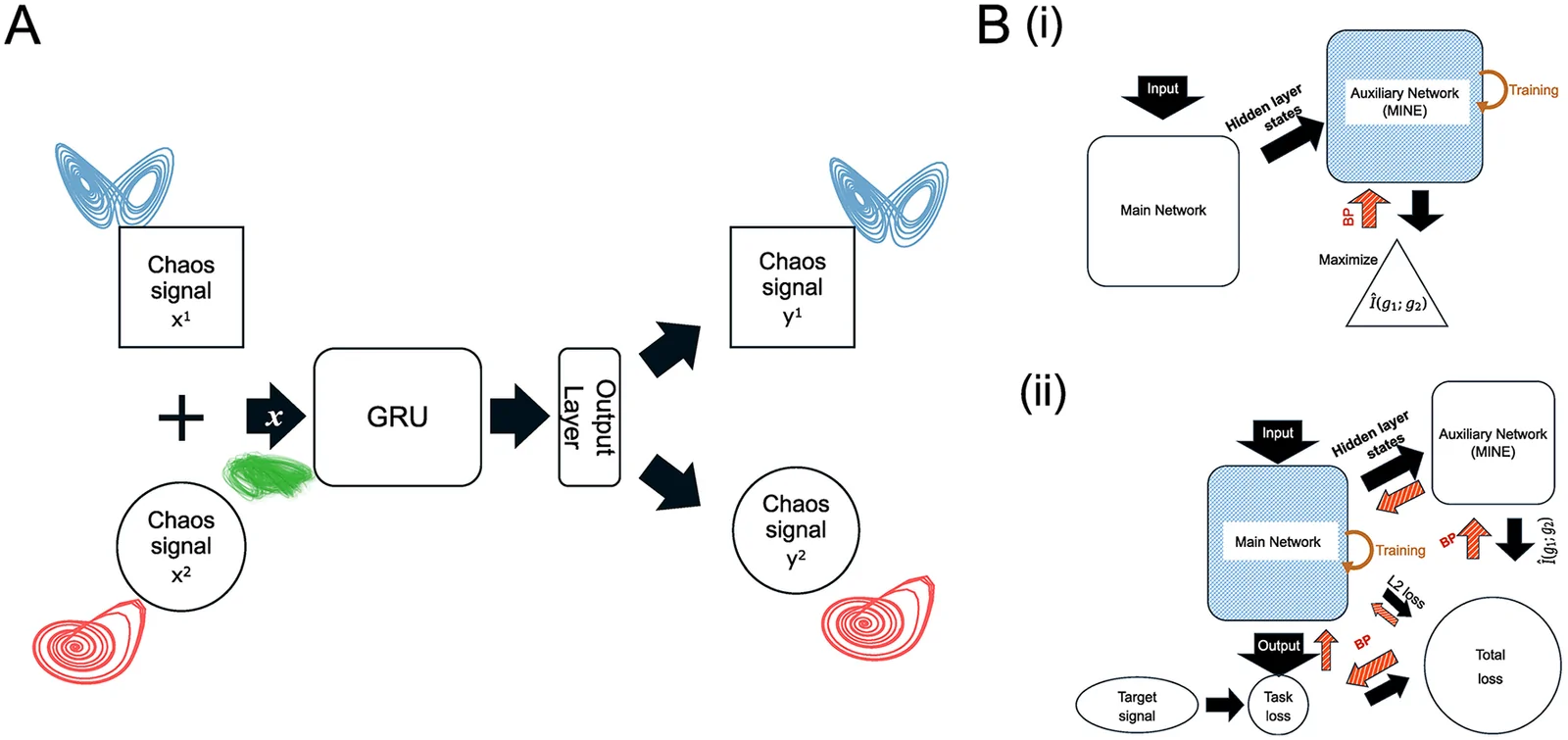
Task and training scheme. **(A)** Chaotic signal separation task. The network receives a superimposed input from the Lorenz and Rössler systems and is required to separate the mixture into two three-dimensional output channels, each reconstructing one of the source signals. **(B)** Training scheme. Two networks are optimized in an alternating, adversarial fashion: the *main network* (the GRU that performs the separation task) and the *auxiliary network* (a MINE estimator of the mutual information between subgroup activities), labeled accordingly in the figure. **(i)** Auxiliary-network training: with the main-network parameters held fixed, the auxiliary network is updated to maximize the estimated mutual information Î(*g*_1_; *g*_2_), yielding an increasingly tight lower bound on the true MI. **(ii)** Main-network training: with the auxiliary-network parameters held fixed, the main network is updated to minimize the total loss combining task error, the estimated MI, and the *L*_2_ regularizer (Equation 6), so that task performance and inter-subgroup statistical independence are optimized simultaneously.

The network receives a superimposed three-dimensional input signal $x_{t}=x_{t}^{\left(1\right)}+x_{t}^{\left(2\right)}$, where $x_{t}^{\left(1\right)}$ and $x_{t}^{\left(2\right)}$ are trajectories generated by the Lorenz and Rössler systems, respectively. The task requires the network to separate this mixture into two distinct three-dimensional output channels, $y_{t}^{\left(1\right)}$ and $y_{t}^{\left(2\right)}$, that reconstruct each original time series (Figure 1A). Task performance was evaluated using the coefficient of determination (*R*^2^) between network outputs and target signals.

### 2.2 Network architecture

We employed a gated recurrent unit (GRU) network (Cho et al., 2014) with *N* = 100 hidden units. The hidden units were divided into two fixed subgroups of equal size: *g*_1_ = {1, …, *N*/2} and *g*_2_ = {*N*/2+1, …, *N*}. This partition defines the populations between which mutual information is minimized (see below); it is not enforced by the readout architecture, so any functional specialization aligned with the subgroups is emergent rather than hardwired. The dynamics of the GRU are given by


$$\begin{array}{ll} {\text{r}}_{t} & =\sigma \left(W_{r}{\text{x}}_{t}+U_{r}{\text{h}}_{t-1}\right), \end{array}$$ (1)

$$\begin{array}{ll} {\text{z}}_{t} & =\sigma \left(W_{z}{\text{x}}_{t}+U_{z}{\text{h}}_{t-1}\right), \end{array}$$ (2)

$$\begin{array}{ll} {\tilde{\text{h}}}_{t} & =\tanh\left(W{\text{x}}_{t}+U\left({\text{r}}_{t}⊙{\text{h}}_{t-1}\right)\right), \end{array}$$ (3)

$$\begin{array}{ll} {\text{h}}_{t} & =\left(1-{\text{z}}_{t}\right)⊙{\text{h}}_{t-1}+{\text{z}}_{t}⊙{\tilde{\text{h}}}_{t}, \end{array}$$ (4)

where *r*_*t*_ is the reset gate, *z*_*t*_ is the update gate, ${\tilde{h}}_{t}$ is the candidate activation, σ is the logistic sigmoid, and ⊙ denotes the Hadamard product. The input signal is three-dimensional ($x_{t}\in {\mathbb{R}}^{3}$) and the hidden state is $h_{t}\in {\mathbb{R}}^{N}$, so the input weight matrices $W_{r},W_{z},W\in {\mathbb{R}}^{N\times 3}$ and the recurrent weight matrices $U_{r},U_{z},U\in {\mathbb{R}}^{N\times N}$ are the trainable parameters of the recurrent layer.

The two output channels are produced by separate linear readouts, each a weighted sum over the entire hidden state:


$$\begin{array}{l} {\text{y}}_{t}^{\left(k\right)}=W_{\text{out}}^{\left(k\right)}{\text{h}}_{t}+{\text{b}}^{\left(k\right)},\;k\in \left\{1,2\right\}, \end{array}$$ (5)

where $W_{\text{out}}^{\left(k\right)}\in {\mathbb{R}}^{3\times N}$ is the readout matrix for channel *k* and *b*^(*k*)^∈ℝ^3^ is a bias. Both readouts thus have access to all *N* units, and no architectural restriction ties a subgroup to a particular output channel; whether the subgroups become specialized for distinct outputs is therefore an emergent consequence of the MI constraint described next.

### 2.3 Training with mutual information minimization

The training objective combines task performance with an information-theoretic constraint:


$$\begin{array}{l} L=L_{\text{task}}+\lambda_{I}Î\left(g_{1};g_{2}\right)+\lambda_{\text{reg}}L_{\text{reg}}, \end{array}$$ (6)

where $L_{\text{task}}$ is the mean squared error between outputs and targets, Î(*g*_1_; *g*_2_) is the estimated mutual information between the activities of the two neural subgroups, and $L_{\text{reg}}$ is an *L*_2_ regularization term. The hyperparameters λ_*I*_ and λ_reg_ control the strength of MI minimization and regularization, respectively.

The mutual information between subgroups was estimated using MINE (Belghazi et al., 2018), which employs an auxiliary neural network to approximate the Donsker-Varadhan representation (Donsker and Varadhan, 1983) of the Kullback-Leibler divergence between the joint distribution of subgroup activities and the product of their marginals. Training alternates between two phases: the MINE network is updated to maximize the MI estimate, while the main network is updated to minimize the total loss including the MI term. This adversarial scheme (Figure 1B) enables simultaneous optimization of task performance and statistical independence between subgroups. For comparison, we trained two additional groups of networks that differ only in their regularization: a group with the MI constraint removed (λ_*I*_ = 0) while retaining the same *L*_2_ regularization, and a second group with neither the MI constraint nor *L*_2_ regularization (λ_*I*_ = λ_reg_ = 0). We refer to the three conditions as *MI*+*L*_2_ (the full model), *L*_2_*-only*, and *unregularized*, respectively; all other architectural and training settings were identical across conditions.

### 2.4 Model selection for analysis

To obtain statistically stable results, training was repeated multiple times with different random initializations of the network weights, generating a population of trained models for each condition. Preliminary experiments showed that models in which a clear internal structure emerged also reached high test performance, whereas insufficiently trained models rarely developed an interpretable structure and yielded unstable evaluation metrics, complicating the interpretation of functional differentiation. We therefore restricted the differentiation and robustness analyses to models whose task performance was assured, while still reporting the overall task accuracy across all runs to characterize learnability. Specifically, for each model we computed the mean coefficient of determination over the six test output dimensions (three for the Lorenz system and three for the Rössler system), ${\bar{R}}^{2}=\frac{1}{6}\overset{6}{\underset{d=1}{\sum }}R_{d}^{2}$, and retained only models satisfying ${\bar{R}}^{2}>0.997$ for the subsequent analyses. The same selection criterion was applied to all training conditions. For each condition, the number of training runs, the number passing this criterion, the acceptance rate, and the ${\bar{R}}^{2}$ distribution before and after selection are reported in Supplementary Table S1; the resulting sample sizes are *N* = 21 (MI+*L*_2_), 36 (*L*_2_-only), and 50 (unregularized).

### 2.5 Evaluation of modularity

**Figure 2:**
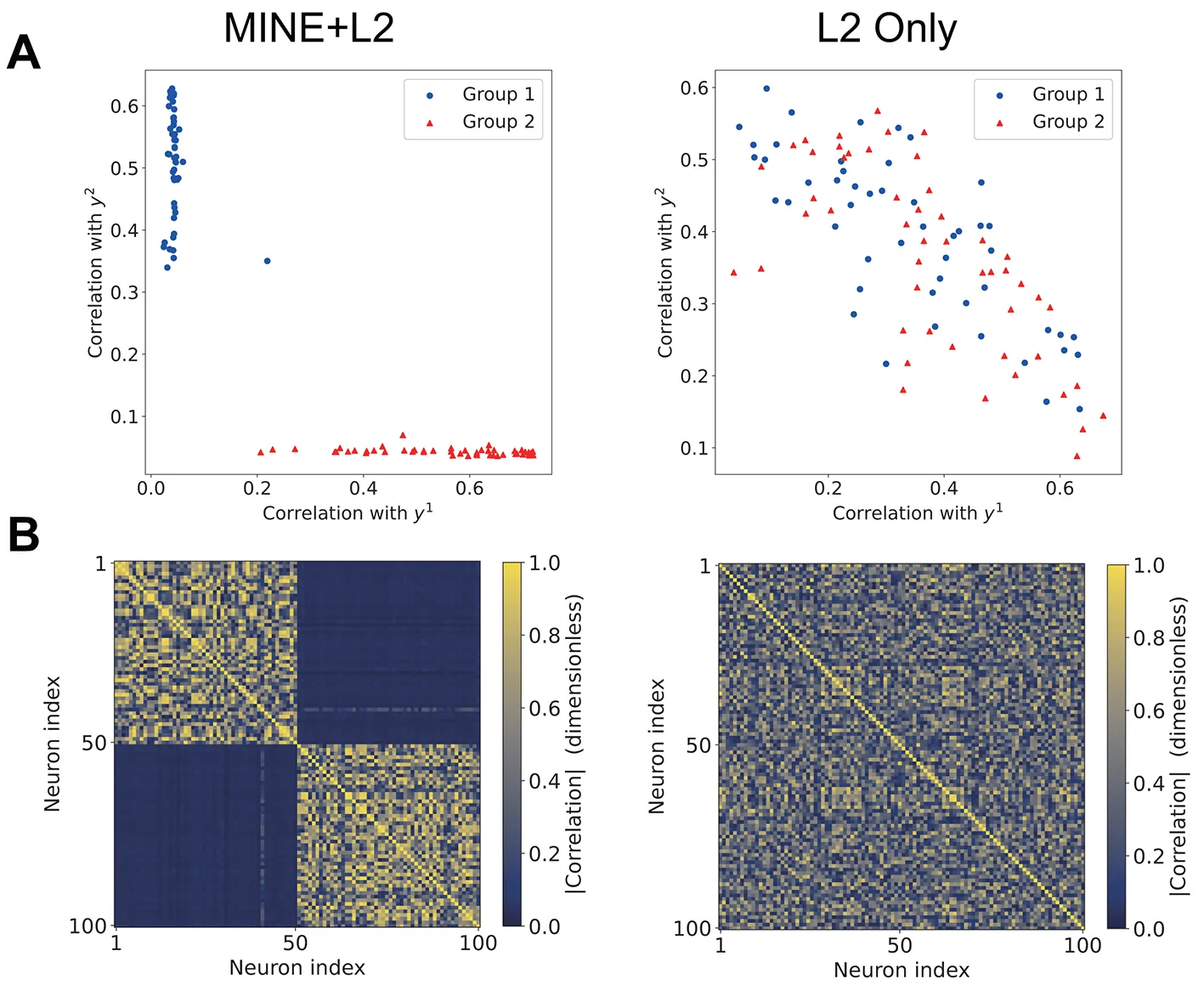
Emergence of functional differentiation under MI minimization. In every panel the left column is the MI+*L*_2_ condition (the full model) and the right column is the *L*_2_-only condition (no MI constraint). **(A)** Per-neuron correlation with each reconstructed output channel (Lorenz vs. Rössler); each point is one neuron, colored by subgroup. Under MI+*L*_2_ the two subgroups separate along the two output axes, whereas under *L*_2_-only they remain intermixed. **(B)** Absolute correlation matrix of GRU activities (matrix of |corr| between neuron pairs), with neurons ordered by subgroup. A block structure aligned with *g*_1_ and *g*_2_ is evident under MI+*L*_2_ but absent under *L*_2_-only.

To characterize the functional organization that emerges under the MI constraint, we analyzed the correlation structure of neural activities following Tomoda et al. (2026). For each neuron we computed the mean absolute correlation between its activity and each of the two reconstructed output signals, yielding a per-neuron selectivity map (Figure 2A). We also computed the full absolute correlation matrix of GRU activities (Figure 2B), in which functionally segregated subgroups appear as a block structure. Both analyses were carried out for the MI+*L*_2_ and *L*_2_-only conditions to compare differentiation with and without the MI constraint.

To characterize the statistical dependence between the two populations more directly, we additionally computed, for every condition, three indicators (Supplementary Table S2): the mean absolute inter-subgroup correlation, taken as the mean of the off-diagonal *g*_1_–*g*_2_ block of the absolute correlation matrix of GRU activities; the final estimated inter-subgroup mutual information Î(*g*_1_; *g*_2_) from the MINE network (available for MI-trained networks); and the segregation of the readout weights, measured for each output as the fraction of its total absolute readout weight that falls on its primary subgroup (0.5 indicating no segregation and 1 complete segregation), averaged over the six output dimensions. Each indicator is reported as a per-condition mean with a 95% confidence interval.

### 2.6 Robustness analysis

We evaluated network robustness through two complementary approaches.

#### 2.6.1 Noise robustness

To assess resistance to input perturbations, we added independent Gaussian noise $\xi_{t}\sim N\left(0,\sigma^{2}I\right)$ to the input signal during testing, sweeping the noise level σ from 0 to 0.3 in steps of 0.01. Each input dimension was min-max normalized to the range [0, 1], so that σ is expressed on the same scale as the input signal (a value of σ = 0.1 corresponds to noise with a standard deviation of 10% of the input range). For each test condition, the coefficient of determination *R*^2^ was computed between network outputs and target signals, averaged over 20 noise realizations to suppress sampling variability within a single trained model. Note that *R*^2^ <0, which can occur at high noise levels, simply indicates a prediction worse than the constant target mean, as expected once the injected noise dominates the signal. We compared all three training conditions defined above (MI+*L*_2_, *L*_2_-only, and unregularized) by aggregating the *R*^2^ curves across the selected models of each condition (Supplementary Table S1). At each σ we tested for differences using Welch *t*-tests for all three pairwise comparisons (MI+*L*_2_ vs. *L*_2_-only, MI+*L*_2_ vs. unregularized, and *L*_2_-only vs. unregularized), with Holm correction applied across the resulting family of tests to control the family-wise error rate. To characterize the whole degradation curve rather than individual noise levels, we additionally computed three per-model, curve-level metrics: the area under the *R*^2^(σ) curve (AUC, by trapezoidal integration over σ), the degradation slope (the least-squares slope of *R*^2^ against σ), and the noise level at which *R*^2^ first fell below a fixed threshold (0.9 and 0.5), obtained by linear interpolation. For each metric we report the per-condition mean with a 95% confidence interval, and for each pairwise comparison the effect size (Cohen's *d*) and the difference with its 95% CI together with a Holm-corrected Welch *t*-test (Supplementary Table S3).

#### 2.6.2 Damage robustness

To examine responses to neuronal loss, we performed ablation experiments by clamping the activities of *k* selected hidden units to zero throughout the test sequence and varying *k* from 0 (the unablated baseline) to 20. For each output channel we partitioned the hidden units into a *primary* and a *non-primary* subgroup, defined as the predefined subgroup with the higher and the lower mean absolute correlation with that output, respectively (using the per-neuron correlations of Section 2.5). Ablation targets a subgroup as a whole: within the targeted subgroup, the *k* units were selected by uniform random sampling without replacement, and, for each *k*, performance was averaged over 20 independent random draws (a fixed random seed was used so that the same masks were applied to every network). No per-unit ranking was used; the only ranking is the subgroup-level primary/non-primary assignment defined above. This labeling was applied to both the MI+*L*_2_ and the *L*_2_-only networks. In MI+*L*_2_ networks the correlation difference between the two subgroups is large, reflecting strong differentiation, whereas in *L*_2_-only networks it is only at the level of fluctuations; nonetheless the same rule assigns a primary and a non-primary subgroup in every network, allowing the two models to be compared under identical ablation protocols. Crossing the two training conditions with the two ablation targets yields four conditions: MI+*L*_2_ primary-ablated, MI+*L*_2_ non-primary-ablated, *L*_2_-only primary-ablated, and *L*_2_-only non-primary-ablated. For each condition and value of *k*, performance was measured separately for the Lorenz and Rössler outputs by their respective *R*^2^ and aggregated across the selected models of each condition. To quantify the selectivity of damage, we computed, per model, the primary-vs.-non-primary ablation gap $g\left(k\right)=R_{\text{non-primary}}^{2}\left(k\right)-R_{\text{primary}}^{2}\left(k\right)$, and report its mean with a 95% confidence interval, comparing conditions at the maximum *k* by Cohen's *d* and a Welch *t*-test. We further fit a linear model *R*^2^~condition × target × *k* and examined the condition-by-target-by-*k* interaction, which tests whether the dependence of damage on the ablation target and its magnitude differs between training conditions (Supplementary Table S4).

## 3 Results

### 3.1 Task performance and functional differentiation

Networks trained with MI minimization successfully learned the chaotic signal separation task. Averaged over all training runs, task accuracy was high in every condition (mean ${\bar{R}}^{2}=0.99$, 0.998, and 0.9997 for MI+*L*_2_, *L*_2_-only, and unregularized, respectively; Supplementary Table S1), confirming that the task was reliably learnable across conditions. Because the MI constraint trades off against peak reconstruction accuracy, the MI+*L*_2_ networks nonetheless reached the strict selection criterion (${\bar{R}}^{2}>0.997$) less often than the controls: 21 of 107 MI+*L*_2_ networks passed (20%), compared with 36 of 50 for *L*_2_-only (72%) and 50 of 50 for unregularized (100%); the corresponding sample sizes and ${\bar{R}}^{2}$ distributions before and after selection are reported in Supplementary Table S1. All selected networks reached essentially saturated accuracy (${\bar{R}}^{2}\approx 0.998$ or above). The differentiation and robustness analyses reported below were performed on these selected networks (*N* = 21, 36, and 50, respectively).

Analysis of neural activity patterns revealed clear functional differentiation under MI minimization. In the MI+*L*_2_ condition, the per-neuron selectivity map (Figure 2A, left) shows that neurons in one subgroup correlated strongly with the Rössler output and weakly with the Lorenz output, while neurons in the other subgroup showed the opposite pattern. The *L*_2_-only condition (Figure 2A, right) instead displayed mixed selectivity, with neurons from both subgroups responding similarly to both output channels. Consistently, the absolute correlation matrix of GRU activities exhibited a pronounced block structure aligned with the predefined subgroups under MI+*L*_2_ (Figure 2B, left), whereas no such block structure was apparent in the *L*_2_-only condition (Figure 2B, right), confirming that the MI constraint, rather than regularization alone, induces functionally segregated subnetworks. The developmental ordering of functional and structural modularity was characterized in our previous study (Tomoda et al., 2026) and is not revisited here.

### 3.2 Noise robustness

**Figure 3:**
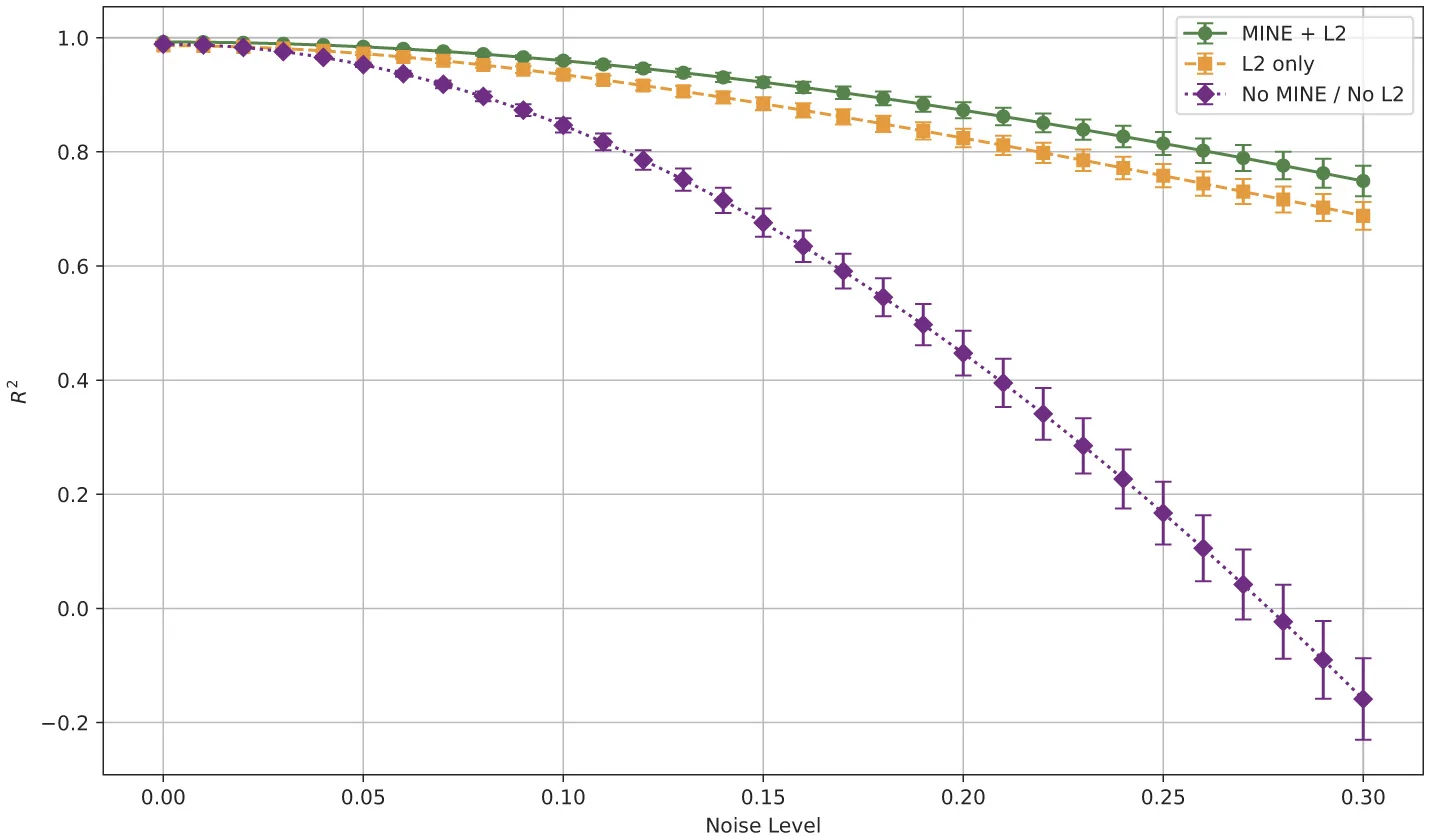
Noise robustness. Reconstruction performance *R*^2^ as a function of the standard deviation σ of input Gaussian noise, swept from 0 to 0.3 in steps of 0.01, for three training conditions: MI minimization with *L*_2_ regularization (MI+*L*_2_; green circles, solid line), *L*_2_ regularization only (λ_*I*_ = 0; yellow squares, dashed line), and neither MI minimization nor *L*_2_ regularization (λ_*I*_ = λ_reg_ = 0; purple diamonds, dotted line). Markers and lines show the across-model mean over the selected networks of each condition (Supplementary Table S1), and error bars indicate the 95% confidence interval across models. Performance is most robust for MI+*L*_2_ and least robust for the unregularized condition; all three pairwise differences are significant at every σ (Welch *t*-test with Holm correction, corrected *p* < 0.05).

We evaluated how training affects resistance to input noise by comparing *R*^2^ across noise levels for the three training conditions (Figure 3). All conditions started from comparably high accuracy in the noise-free case (*R*^2^>0.997 at σ = 0), so any separation between the curves at higher noise levels reflects a genuine difference in how rapidly performance degrades rather than differences in baseline accuracy. The three conditions formed a clear ordering across the entire σ range tested (0–0.3): the MI+*L*_2_ networks (green) decayed most slowly and were the most robust, the *L*_2_-only networks (yellow) were intermediate, and the unregularized networks (purple) degraded fastest and were the least robust. Per-σ Welch *t*-tests with Holm correction across the three pairwise comparisons confirmed that these differences were significant at every noise level examined (corrected *p* < 0.05). Curve-level metrics quantified this ordering (Supplementary Table S3). The area under the *R*^2^(σ) curve was largest for MI+*L*_2_ (0.272, 95% CI [0.268, 0.274]), intermediate for *L*_2_-only (0.261, [0.257, 0.264]), and smallest for unregularized (0.177, [0.168, 0.185]); the MI+*L*_2_ advantage over *L*_2_-only was statistically robust with a large effect size (Cohen's *d* = 1.17, Holm-corrected *p* = 1.7 × 10^−5^), and all three pairwise comparisons were significant. Consistently, the degradation slope was shallowest for MI+*L*_2_ (−0.83 vs. −1.04 for *L*_2_-only and −3.89 for unregularized), and the noise level at which *R*^2^ first fell below 0.9 was highest for MI+*L*_2_ (σ = 0.178 vs. 0.141 and 0.081). The separation between MI+*L*_2_ and *L*_2_-only isolates the contribution of MI minimization beyond regularization, while the gap between *L*_2_-only and unregularized reflects the additional benefit of *L*_2_ regularization; together they indicate that MI minimization and regularization make complementary contributions to noise-tolerant internal representations.

### 3.3 Damage robustness

**Figure 4:**
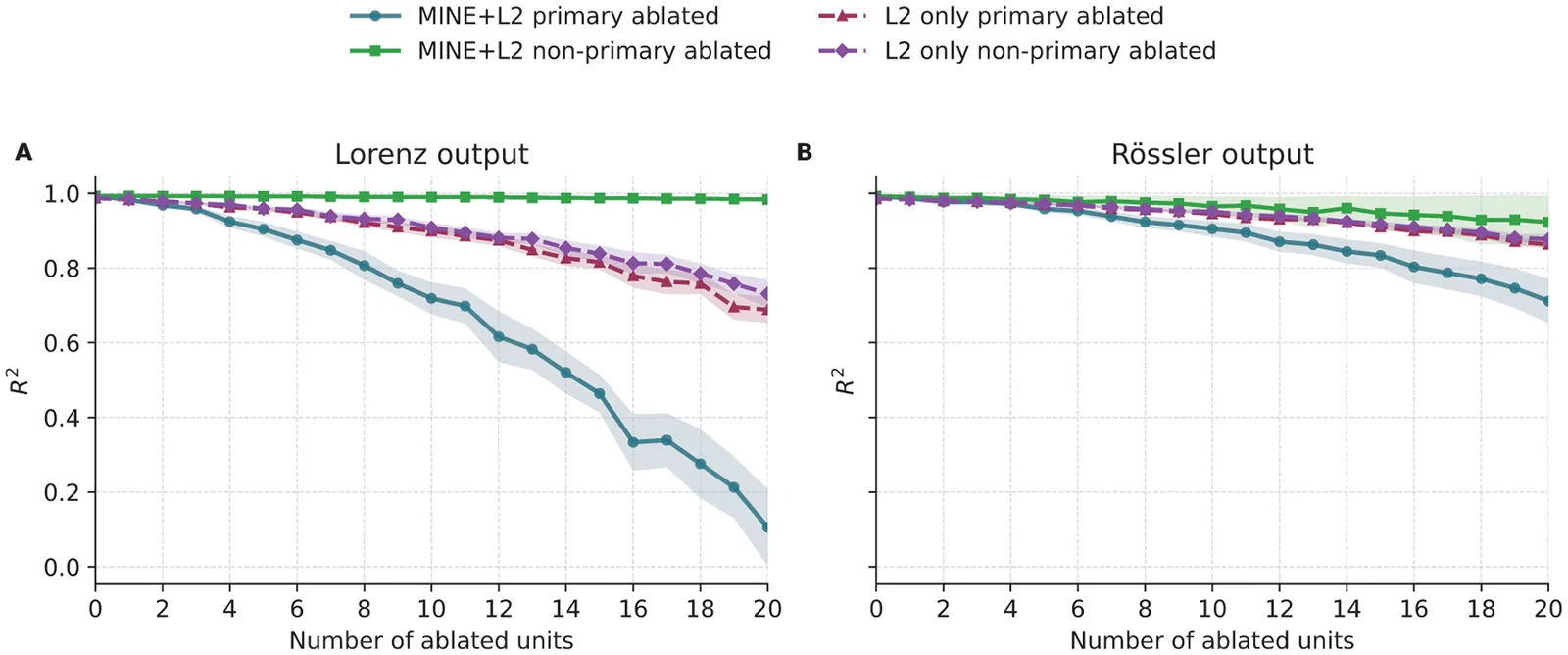
Damage robustness under selective ablation. Reconstruction performance *R*^2^ as a function of the number *k* of ablated hidden units, shown separately for **(A)** the Lorenz output (left panel) and **(B)** the Rössler output (right panel). For each output, the hidden units of every network are split into a primary and a non-primary subgroup (the predefined subgroup with the higher and lower mean correlation with that output, respectively), and neurons are ablated from one subgroup at a time. Each panel overlays four conditions: MI+*L*_2_ primary-ablated (blue circles, solid line), MI+*L*_2_ non-primary-ablated (green squares, solid line), *L*_2_-only primary-ablated (brown triangles, dashed line), and *L*_2_-only non-primary-ablated (purple diamonds, dashed line). Markers and lines show the across-model mean over the selected networks of each condition (Supplementary Table S1), and shaded bands indicate the 95% confidence interval across models. In MI+*L*_2_ networks, ablation is highly localized, with a steep decline when the primary subgroup is removed but almost none when the non-primary subgroup is removed, whereas the weakly differentiated *L*_2_-only network shows only a small difference between the two ablation targets.

Ablation experiments revealed a strongly direction-dependent damage response that distinguished the two training conditions (Figure 4; the same pattern held for both the Lorenz and Rössler outputs, shown in the left and right panels). The four conditions formed a consistent ordering of *R*^2^ for both outputs: MI+*L*_2_ non-primary-ablated > *L*_2_-only non-primary-ablated ≳ *L*_2_-only primary-ablated > MI+*L*_2_ primary-ablated. In the MI+*L*_2_ network, ablating the primary subgroup of an output degraded that output steeply with the number of ablated units *k*, whereas ablating its non-primary subgroup left the output essentially intact across the full range of *k*. This large separation between primary and non-primary ablation is the central signature of functional localization: in a differentiated network, damage matters almost entirely in proportion to how strongly the ablated population is coupled to the output. By contrast, the *L*_2_-only network showed only a small gap between its primary- and non-primary-ablation curves, consistent with its near-absence of differentiation; removing either subgroup degraded the output by a comparable, intermediate amount. We quantified this selectivity by the primary-vs.-non-primary ablation gap at the maximum *k* (Supplementary Table S4). For the Lorenz output the gap was 0.878 (95% CI [0.774, 0.982]) in MI+*L*_2_ networks vs. 0.043 ([−0.024, 0.109]) in *L*_2_-only networks (Cohen's *d* = 4.0, *p* = 2 × 10^−16^); for the Rössler output it was 0.211 ([0.085, 0.337]) vs. 0.014 ([−0.006, 0.033]) (*d* = 1.1, *p* = 0.004). A linear model of *R*^2^ on training condition, ablation target, and number of ablated units confirmed a significant condition-by-target-by-*k* interaction for both outputs (*p* < 10^−40^), establishing that damage depends on the ablation target far more steeply in MI+*L*_2_ than in *L*_2_-only networks.

Together, these results demonstrate that the functional differentiation induced by MI minimization leads to localized damage effects: targeted removal of a specialized subgroup is costly for its associated output, whereas damage that spares that subgroup leaves the corresponding function essentially intact. The weakly differentiated *L*_2_-only network does not exhibit this separation.

## 4 Discussion

The present study demonstrates that minimizing mutual information between neural subgroups not only induces functional differentiation but also enhances network robustness. While our previous work (Tomoda et al., 2026) established that MI minimization promotes the emergence of functionally specialized modules in recurrent neural networks, the current findings reveal an additional benefit: differentiated networks exhibit superior resistance to input noise and qualitatively distinct responses to neuronal damage. These results suggest that functional specialization serves an adaptive role beyond mere computational efficiency.

The enhanced noise robustness observed in differentiated networks can be attributed to the statistical independence between subgroups. Direct measurements support this interpretation (Supplementary Table S2): the mean absolute correlation between the two subgroups' activities, and the final estimated inter-subgroup MI, were much lower in MI+*L*_2_ networks than in the *L*_2_-only and unregularized controls, and the readout weights were correspondingly more segregated across subgroups. When MI between subgroups is minimized, noise-driven fluctuations in one subgroup are thus less likely to be shared with the other. Because the readouts draw on the full hidden state, this reduced cross-talk lowers the interference that noise in one population imposes on the output associated with the other. This interpretation aligns with theoretical proposals that modular organization limits the spread of perturbations within complex systems, keeping the effects of local disturbances largely confined to the affected module (Simon, 1962; Sporns and Betzel, 2016). In information-theoretic terms, the MINE-based constraint can be viewed as imposing a representational bottleneck that decouples task-relevant information across populations, a perspective conceptually related to the information bottleneck framework for deep networks (Tishby and Zaslavsky, 2015), whose empirical claims nonetheless remain debated (Saxe et al., 2019).

The ablation experiments revealed that functional differentiation fundamentally alters how the network responds to damage. Within the MI+*L*_2_ network, removing neurons from the subgroup primarily coupled to a given output degraded that output steeply, whereas removing the weakly coupled subgroup left it almost intact, showing that the two functions have become largely separable and each depends on a dedicated population. The weakly differentiated *L*_2_-only network, in which the two subgroups are distinguished only at the level of fluctuations, instead degraded by a similar intermediate amount whichever subgroup was ablated. The large primary-vs.-non-primary gap appears only under MI minimization, not under *L*_2_ regularization alone, indicating that the localized damage response is a direct consequence of the functional partition rather than of generic capacity or trainability. These patterns carry two complementary implications: targeted damage to a specialized population can severely impair its function; conversely, damage that spares that population leaves the corresponding function essentially intact. This is a form of fault containment, in which damage stays confined to the affected function, and it is absent in the weakly differentiated control network. This trade-off may explain why biological neural systems exhibit both distributed and localized representations depending on computational demands (Rigotti et al., 2013).

At the level of the present model, these findings offer a possible computational principle relevant to theoretical proposals on the adaptive significance of brain modularity (Kashtan and Alon, 2005; Clune et al., 2013): that functionally differentiated representations, induced here by an information-theoretic constraint, can support perturbation containment. The results are thus consistent with the idea that functional modularity may help maintain reliable function in noisy environments and under partial damage; we emphasize that this is a computational demonstration in an artificial network rather than direct evidence about biological evolution. This adaptive benefit complements the developmental observation from our previous study (Tomoda et al., 2026), in which functional modularity emerged before structural modularity, together suggesting that activity-dependent specialization both precedes structural reorganization and yields tangible robustness advantages. The dissociation between functional and structural organization also resonates with recent work showing that structural modularity need not entail functional specialization, and that specialization is favored when task features are separable and network resources are constrained (Béna and Goodman, 2025); our setting, with two clearly separable chaotic sources and a regularized training regime, provides precisely such conditions, while the MI constraint additionally supplies an explicit information-theoretic pressure toward segregation.

The MI constraint does carry a modest cost in trainability: MI+*L*_2_ networks reached the strict ${\bar{R}}^{2}>0.997$ selection criterion far less frequently than the controls (20% vs. 72% and 100%; Supplementary Table S1), reflecting a trade-off between minimizing inter-subgroup information and maximizing peak reconstruction accuracy. Because all analyses were performed on networks matched at essentially saturated noise-free accuracy (the three conditions overlap at σ = 0), the differences reported here emerge only as noise or damage is introduced, rather than from differences in baseline performance.

Several limitations should be noted, which also mark natural directions for future work. First, we deliberately studied the minimal case that isolates the effect: two predefined subgroups and two clearly separable sources, which maps onto a two-module solution. Two questions follow. (i) The relationship between the number of predefined subgroups and the number of sources: settings in which the groups do not match, or greatly outnumber, the sources are biologically more realistic and would test whether MI minimization still yields useful specialization when the partition is not aligned with the task. A natural extension is to more than two subgroups with pairwise MI penalties. (ii) Harder separation problems. Lorenz and Rössler differ markedly, so the sources already share little mutual information; separating same-class sources, such as two Lorenz systems with slightly different parameters, would be more demanding and would more stringently test the value of MI minimization. Second, the ablation experiments involved complete removal of neural activity, whereas biological damage often produces more graded effects; future work should examine robustness under more realistic damage models. Finally, extending this framework to spiking neural networks would strengthen the biological relevance of our findings.

## Data Availability

The code used to generate and analyze the simulations of this study is openly available on GitHub at https://github.com/yymgch/mirnn-robust and archived at Zenodo (DOI: https://doi.org/10.5281/zenodo.21287687).
