# Supplementary material for: Mutual information minimization enhances noise robustness and induces fault containment in functionally differentiated recurrent neural networks
Source: Front Neurosci. 2026 Aug 13;20:1892212. doi: 10.3389/fnins.2026.1892212 (PMC13518512; doi:10.3389/fnins.2026.1892212)
Supplement: Supplementary file 1 [file Data_Sheet_1.pdf]

Supplementary Material

1 SUPPLEMENTARY TABLES

All values below were obtained from the networks trained anew for this study (each trained for 100 epochs of 100 minibatch updates, i.e. 10^4 gradient steps). Models were selected for the differentiation and robustness analyses by the criterion $\bar{R}^2 > 0.997$ (mean coefficient of determination over the six output dimensions), evaluated on 500 held-out test sequences.

Table S1. Model selection flow. For each training condition: the number of independently initialized training runs, the mean $\bar{R}^2$ across all runs (before selection; characterizing learnability), the number passing the $\bar{R}^2 > 0.997$ selection criterion, the corresponding acceptance rate, the mean $\bar{R}^2$ of the selected networks, and the sample size N used in the differentiation and robustness analyses. Task accuracy across all runs is high in every condition, confirming learnability; the lower pass rate for MI+L_2 reflects the trade-off between the mutual-information constraint and peak reconstruction accuracy (its larger before-selection spread stems from occasional non-converged runs). Before-selection statistics are computed over the runs with a recorded $\bar{R}^2$.

Condition	Runs	$\bar{R}^2$ (all runs)	Passing $\bar{R}^2 > 0.997$	Rate	$\bar{R}^2$ (selected)	N
MI+L_2	107	0.987 ± 0.047	21	20%	0.998	21
L_2-only	50	0.998 ± 0.001	36	72%	0.999	36
unregularized	50	0.9997 ± 0.0002	50	100%	0.9997	50

Table S2. Mechanism indicators across conditions (mean with 95% CI across the selected networks). *Inter-subgroup* $|\mathrm{corr}|$: the mean absolute correlation between the activities of the two predefined subgroups (mean of the off-diagonal g_1–g_2 block of the absolute correlation matrix of GRU activities). *Final MINE MI* $\hat{I}(g_1; g_2)$: the final estimate of the inter-subgroup mutual information from the MINE network (defined only for the MI-trained condition). *Readout primary-weight fraction*: for each output, the fraction of its total absolute readout weight that falls on its primary subgroup (0.5 = no segregation, 1 = complete segregation), averaged over the six outputs. MI+L_2 networks show markedly reduced inter-subgroup dependence and more segregated readouts, supporting the reduced-cross-talk interpretation.

Indicator	MI+L_2	L_2-only	unregularized
Inter-subgroup $\lvert\mathrm{corr}\rvert$	0.056 [0.037, 0.076]	0.373 [0.370, 0.375]	0.468 [0.463, 0.474]
Final MINE MI $\hat{I}(g_1; g_2)$	0.010 [0.007, 0.012]	—	—
Readout primary-weight fraction	0.887 [0.876, 0.899]	0.536 [0.532, 0.539]	0.524 [0.522, 0.526]

Table S3. Curve-level noise-robustness metrics. Per-model metrics of the $R^2(\sigma)$ degradation curve (mean with 95% confidence interval across models): area under the curve (AUC), least-squares degradation slope, and the noise level σ at which R^2 first falls below 0.9. Pairwise comparisons of the AUC (Cohen's d; Welch t-test, Holm-corrected): MI+L_2 vs L_2-only $d = 1.17$, $p = 1.7 \times 10^{-5}$; MI+L_2 vs unregularized $d = 3.74$, $p = 5.7 \times 10^{-29}$; L_2-only vs unregularized $d = 3.55$, $p = 4.6 \times 10^{-27}$.

Metric	MI+L_2	L_2-only	unregularized
AUC	0.272 [0.268, 0.274]	0.261 [0.257, 0.264]	0.177 [0.168, 0.185]
Degradation slope	-0.83 [-0.93, -0.74]	-1.04 [-1.13, -0.96]	-3.89 [-4.14, -3.64]
σ at $R^2 < 0.9$	0.178 [0.166, 0.191]	0.141 [0.133, 0.148]	0.081 [0.078, 0.084]

Table S4. Selective-ablation statistics. The primary-versus-non-primary ablation gap $g = R^2_{\text{non-primary}} - R^2_{\text{primary}}$ at the maximum number of ablated units ($k = 20$), per output and condition (mean with 95% CI), with the between-condition comparison (Cohen's d; Welch t-test). A linear model $R^2 \sim$ condition $\times$ target $\times$ k yielded a highly significant condition-by-target-by-k interaction for both outputs (Lorenz $p < 10^{-200}$; Rössler $p = 2 \times 10^{-46}$).

Output	MI+L_2 gap	L_2-only gap	Cohen's d	p
Lorenz	0.878 [0.774, 0.982]	0.043 [-0.024, 0.109]	4.0	2×10^{-16}
Rössler	0.211 [0.085, 0.337]	0.014 [-0.006, 0.033]	1.14	0.004
